# Effects of autism spectrum disorders on outcome in teenage-onset anorexia nervosa evaluated by the Morgan-Russell outcome assessment schedule: a controlled community-based study

**DOI:** 10.1186/s13229-015-0013-4

**Published:** 2015-03-08

**Authors:** Søren Nielsen, Henrik Anckarsäter, Carina Gillberg, Christopher Gillberg, Maria Råstam, Elisabet Wentz

**Affiliations:** Department of Child and Adolescent Psychiatry, Region Sjælland, Child Psychiatric Clinic, Næstved, Denmark; Forensic Psychiatry, Institute of Neuroscience and Physiology, University of Gothenburg, Göteborg, Sweden; Gillberg Neuropsychiatry Centre, Institute of Neuroscience and Physiology, University of Gothenburg, Kungsgatan 12, SE- 411 19 Göteborg, Sweden; Department of Child and Adolescent Psychiatry, Strathclyde University, Yorkhill Hospital, Glasgow, UK; Institute of Child Health, University College, London, UK; Department of Clinical Sciences, Child and Adolescent Psychiatry, Lund University, Lund, Sweden

**Keywords:** Anorexia nervosa, Autism spectrum disorders, Long-term outcome, Psychosexual, Psychosocial, Community-based, Prospective, Controlled

## Abstract

**Background:**

The purpose of the study was to evaluate time trends and effects of co-existing autism spectrum disorders (ASD) on outcome in an ongoing long-term follow-up study of anorexia nervosa (AN).

**Methods:**

The Morgan-Russell Outcome Assessment Schedule (MROAS) was used at 6-, 10- and 18-year follow-up of a representative sample of 51 individuals with teenage-onset AN and a matched group of 51 healthy comparison cases. The full multinomial distribution of responses for the full scale and each of the subscales was evaluated using exact nonparametric statistical methods. The impact of diagnostic stability of ASD on outcome in AN was evaluated in a dose–response model.

**Results:**

There were no deaths in either group. Food intake and menstrual pattern were initially poor in the AN group but normalised over time. MROAS ‘mental state’ was much poorer in the AN group and did not improve over time. The psychosexual MROAS domains ‘attitudes’ and ‘aims’ showed persistent problems in the AN group. In the MROAS socioeconomic domain, the subscales ‘personal contacts’, ‘social activities’ and ‘employment record’ all showed highly significant between-group differences at all three follow-ups. A statistically significant negative dose–response relationship was found between a stable diagnosis of ASD over time and the results on the subscales ‘mental state’, ‘psychosexual state’ and ‘socio-economic state’.

**Conclusions:**

Outcome of teenage-onset AN is favourable with respect to mortality and persisting eating disorder, but serious problems remain in the domains ‘mental state’, ‘psychosexual function’ and ‘socioeconomic state’. Outcome is considerably worse if ASD is present. Treatment programmes for AN need to be modified so as to accommodate co-existing ASD.

## Background

Anorexia nervosa (AN) is one of the most serious psychiatric disorders that affect teenagers, predominantly girls, with an increased risk of a chronic course and premature death [1-3]. The prevalence of AN is estimated at 0.3% to 2.2% [3,4]. Long-term outcome in eating disorders (EDs) can be assessed in different areas such as mortality, recovery, psychosocial functioning and psychiatric and somatic comorbidity. Discouraging data on psychosocial outcome of AN was reported by Ratnasuriya and co-workers regarding AN patients at 20-year follow-up [5]. Just over half of the individuals did get married, and even fewer had children. Further, only half the group was employed and one third led a very isolated life. Other follow-up studies confirm that former AN patients marry less often [6,7]. Twelve percent of Swedish women with a history of inpatient treatment for AN had a disability pension at long-term follow-up [8].

The first suggestion of a possible relationship between autism spectrum disorder (ASD) and AN came from Christopher Gillberg more than 30 years ago [9]. Since then, a substantial body of work has been published, theoretical [10-14] as well as clinical and epidemiological [15-17]. Our group has recently contributed to this body of evidence [18,19]. In the Gothenburg study, the AN and the healthy comparison (COMP) groups have so far been examined on four occasions at mean age 16, 21, 24 and 32 [20-24]. At the time of the latest examination, 18 years had elapsed since the onset of AN. Screening for ASD has been part of the four examinations of the AN and COMP groups [18,19]. Thirty-two percent of the AN group have, at one or more occasions, been assigned a diagnosis of ASD, and 12% had ASD in all four waves of the study. The individuals with ASD in the AN group had the worst psychosocial outcome at 18-year follow-up [19,25]. The Gothenburg study has also shown that autistic traits pertaining to social interaction are overrepresented among the first-degree relatives of individuals with AN [26].

Earlier work [25,27] prompted us to perform the present study, with the aim of getting more information out of the Morgan-Russell outcome assessment schedule (MROAS) [28,29] than just the average composite score and general outcome categories, which are conventionally the only reported statistics. In particular, we wanted to analyse the influence of diagnostic stability of ASD on outcome as measured by these scales.

## Methods

### Participants

#### Anorexia nervosa group

As a detailed description has been given repeatedly previously [18-25], it should suffice to describe that the AN group consisted of 51 cases of teenage-onset AN. Twenty-three girls and two boys were found via screening of the whole 1970 birth cohort (*N*: 4,291) (1 girl refused in-depth study leaving 22 girls and 2 boys for examination) in Gothenburg, Sweden. This population-based group was supplemented by another community sample, 26 girls and 1 boy, who were referred to the research team primarily by school health staff. The latter group was born mainly in the period 1971 to 1974. All cases fulfilled DSM-III-R [30] and DSM-IV [31] criteria for AN within a year of the assessment of the original study. These two groups combined constituted the AN group.

### Comparison group

For each of the 51 cases with AN, the school nurses selected the same-sexed classmate without a history of an ED closest in age to the index child. These individuals served as healthy controls. Collectively, they were referred to as the COMP group.

### Procedure

At all studies, the AN group and the COMP group were examined using identical test batteries. All 102 individuals (51 AN and 51 COMP cases) were examined at baseline, in AN study 1, at age 16, again in AN study 2, at age 21 (the 6-year follow-up), and in AN study 3, at age 24 (the 10-year follow-up). In AN study 4, 45 out of 51 in the AN group were examined in person; five were interviewed over the telephone; and in one case, the mother was interviewed as she did not want to let the research team have access to her daughter. In the COMP group, all 51 were interviewed, 48 in person, and three over the telephone.

At all four examinations, an investigator blinded to the original AN/COMP group status assigned ASD diagnoses. At the time of AN study 1, all mothers took part in an in-depth interview regarding early development and childhood symptoms and behaviours. On the basis of de-identified case notes concerning premorbid history, the blinded investigator assigned diagnoses of ASD [32]. In each follow-up study [19,25], when only three individuals had persisting underweight, a new blinded investigator assigned ASD diagnoses after personal interview (see Table 1). Those individuals who had never been assigned an ASD diagnosis were classified as ‘never ASD’ (*n* = 34); those who had been assigned an ASD diagnosis in at least one study but not at all four studies were classified as ‘ASD × 1-3’ (*n* = 10), and those with an ASD diagnosis at all four examinations were classified as ‘ASD × 4’ (*n* = 6). One woman in the AN group was not assigned an ASD category; she was first diagnosed with ASD after suffering severe traumatic brain injury at about 20 years of age.

**Table 1:** **Instruments used at AN study 1 to AN study 4 to assess ASD**

	**Type of instrument**
**AN study 1**	Structured interview with mother
**AN study 2**	Dewey social awareness test [42], checklist for autistic disorder [30] and Asperger’s syndrome [43]
**AN study 3**	Asperger Syndrome Diagnostic Interview [44], checklist for autistic disorder [31] and Asperger’s syndrome [43]
**AN study 4**	Asperger Syndrome Diagnostic Interview [44], checklist for autistic disorder [31] and Asperger’s syndrome [43] Autism-Spectrum Quotient [45]

*AN study 1* first anorexia nervosa examination, *AN study 2* second anorexia nervosa examination, *AN study 3* third anorexia nervosa examination, *AN study 4* fourth anorexia nervosa examination, *ASD* autism spectrum disorders.

### Instruments

The Morgan-Russell outcome assessment schedule (MROAS) [28] is a widely used and well-validated instrument, originally designed to enhance good clinical practice, monitor change in the individual patient and facilitate comparisons between treatment centres. The revision suggested by Morgan and Hayward [29] was used in this study. Overall outcome was reported after each follow-up of the Gothenburg study [21,22,25] as Morgan-Russell’s average composite score and as general outcome classification (good, intermediate and poor outcome).

In this particular project, we focus on the responses from each individual on each Morgan-Russell scale and subscale [28,29].

### Ethics

The study was approved by the human ethics committee at the Medical Faculty of University of Gothenburg. The individuals participated voluntarily after informed consent was obtained.

### Data structure

The responses on the Morgan-Russell scales and subscales range from 0 to 12. The individual scales and subscales have three, four or five response categories making the resulting multinomial tables unbalanced with empty cells. In consequence, statistical methods based on normality assumptions cannot be used; and instead, we used exact nonparametric (permutation) statistical methods [33-36]. Computations were performed in the program package StatXact8 [37] on a PC platform.

### Statistical analysis

This study is a longitudinal study. The overall design is a repeated measures analysis. The data structure rules out the use of standard methods like one-way repeated measures analysis of variance.

The set of multinomial tables was analysed using exact nonparametric (permutation) methods. Computationally, these analyses are rather demanding; so in most cases, it is not possible to compute an exact *P* value due to limitations in RAM memory. Consequently, a Monte Carlo option is used. Routinely, the sampling procedure is set to sampling 10,000 tables, which provides an unbiased point estimate and a 99% confidence interval for the true *P* value.

First, we performed analyses of ‘stratified singly ordered row by column (*r* × *c*) contingency tables’. There are three strata, one for each follow-up: AN study 2, 3 and 4. There are two groups: AN and COMP. The analysis is a generalised Cochran-Mantel-Haenszel Test [35]. The *H*_0_ is no association between the *r* rows (AN and COMP) and the *c* columns (response categories) in any of the *s* strata (AN study 2, 3 and 4).

Second, we analysed for group differences at each point in time. The tables are ‘singly ordered *r* × *c* contingency tables’ (the rows (AN or COMP) are unordered, but the columns - the response categories - are ordered), and thus the relevant test is Kruskal-Wallis Test [33]. *H*_0_ the multinomial probabilities across the response categories are identical in the two groups. Results of the statistical analyses can be found in the right side of the raw data in Tables 2, 3 and 4.

**Table 2:** **Morgan-Russell subscale C (mental state); comparisons between AN and COMP group at 6-, 10- and 18-year follow-up**

**Follow-up**	**AN group**				**COMP group**				**Singly ordered r × c contingency tables Kruskal-Wallis test**	**Exact Monte Carlo**
	**Response category**				**Response category**				**Test statistic**	***P*** **(99% CI)**
	**0**	**4**	**8**	**12**	**0**	**4**	**8**	**12**		
6 year	1	10	13	27	0	0	8	43	13.72	0.0001 (0 to 0.0004)
10 year	1	10	14	26	1	2	4	44	13.91	0 (0 to 0.0005)
18 year	2	10	14	25	1	0	7	43	15.43	0.0001 (0 to 0.0005)
Doubly ordered r × c contingency tables	Linear-by-linear association test				Linear-by-linear association test				Stratified r × c contingency tables	
Analysis for time trend									Cochran-Mantel-Haenszel test	
Test statistic	−0.4583				−0.3885				39.72	
Exact Monte Carlo *P*	0.69				0.77				0	
99% CI	0.67 to 0.7				0.76 to 0.78				0 to 0.0005	

*AN* anorexia nervosa, *COMP* comparison, *CI* confidence interval, *r × c* rows and columns.

**Table 3:** **Morgan-Russell subscale E2 (emancipation from family);comparisons between AN and COMP group at 6-, 10- and 18-year follow-up**

**Follow-up**	**AN group**				**COMP group**				**Singly ordered r × c contingency tables Kruskal-Wallis test**	**Exact Monte Carlo**
	**Response category**				**Response category**				**Test statistic**	***P*** **(99% CI)**
	**0**	**4**	**8**	**12**	**0**	**4**	**8**	**12**		
6 year	3	9	9	30	0	2	11	38	4.492	0.032 (0.027 to 0.036)
10 year	2	5	9	35	0	1	12	38	0.948	0.34 (0.33 to 0.35)
18 year	2	3	9	37	0	0	4	47	7.158	0.007 (0.005 to 0.009)
Analysis for time trend doubly ordered r × c contingency tables	Linear-by-linear association test				Linear-by-linear association test				Stratified r × c contingency tables Cochran-Mantel-Haenszel test	
Test statistic	+1.715				+2.378				15.8	
Exact Monte Carlo *P*	0.097				0.022				0	
99% CI	0.09 to 0.104				0.018 to 0.025				0 to 0.0005	

*AN* anorexia nervosa, *COMP* comparison, *CI* confidence interval, *r × c* rows and columns.

**Table 4:** **Morgan Russell subscale E5 (employment record); comparisons between AN and COMP group at 6-, 10- and 18-year follow-up**

**Follow-up**	**AN group**				**COMP group**				**Singly ordered r × c contingency tables Kruskal-Wallis test**	**Exact Monte Carlo**
	**Response category**				**Response category**				**Test statistic**	***P*** **(99% CI)**
	**0**	**4**	**8**	**12**	**0**	**4**	**8**	**12**		
6 year	9	4	5	33	4	3	3	41	3.256	0.077 (0.07 to 0.084)
10 year	8	3	7	33	1	2	3	45	8.245	0.0026 (0.001 to 0.004)
18 year	17	3	7	24	2	2	11	36	9.881	0.001 (0.0002 to 0.002)
Doubly ordered r × c contingency tables Analysis for time trend	Linear-by-linear association test				Linear-by-linear association test				Stratified r × c contingency tables Cochran-Mantel-Haenszel test	
Test statistic	−1.911				0				22.8	
Exact Monte Carlo *P*	0.06				1				0	
99% CI	0.05 to 0.07				0.9995 to 1				0 to 0.0005	

*AN* anorexia nervosa, *COMP* comparison, *CI* confidence interval, *r × c* rows and columns.

Third is the analysis for change over time. Each group is analysed separately. Data is organised as ‘doubly ordered *r* × *c* contingency tables’, so the relevant statistical test is linear-by-linear association test [34]. The rows correspond to the different follow-ups, and the columns are the responses. The *H*_0_ is the identical multinomial probabilities at the three points in time, that is, at AN study 2, 3 and 4. The chosen statistical test is appropriate for detecting departures from the null hypothesis *H*_0_ of the form *H*_1_ or *H′*_1_ or for detecting the two-sided alternative that either *H*_1_ or *H′*_1_ is true. Hypothesis *H*_1_ implies that as we move from one row to the next, the probability of the response falling in the next higher category increases. Hypothesis *H′*_1_ states the opposite. A large *positive* value of the test statistic rejects *H*_0_ in favour of *H*_1_ (that is, a ‘better’ outcome), while a large *negative* value rejects *H*_0_ in favour of *H′*_1_ (that is, a ‘worse’ outcome). The results from this statistical test are located below each group at the bottom of the group-difference tables, that is Tables 2, 3 and 4, and at the right side of the dose–response tables, that is Tables 5, 6 and 7.

**Table 5:** **Morgan-Russell subscale C (mental state); dose–response analysis**

**Dose**	**AN study 2**				**AN study 3**				**AN study 4**				**Analysis for time trend doubly ordered r × c contingency tables Linear-by-linear association test**	**Exact Monte Carlo**
	**Response category**				**Response category**				**Response category**				**Test Statistic**	***P*** **(99% CI)**
	**0**	**4**	**8**	**12**	**0**	**4**	**8**	**12**	**0**	**4**	**8**	**12**		
Never ASD	0	4	12	18	0	5	9	20	0	6	8	20	0	1 (0.9995 to 1)
ASD × 1-3	0	3	1	6	0	1	4	5	0	2	3	5	0	1 (0.9995 to 1)
ASD × 4	1	3	0	2	1	3	1	1	2	1	3	0	−0.561	0.69 (0.67 to 0.7)
Dose–response analysis														
Doubly ordered r × c contingency tables	Linear-by-linear association test				Linear-by-linear association test				Linear-by-linear association test				Stratified r × c contingency tables Cochran-Mantel-Haenszel test	
Test statistic	−1.452				−2.56				−2.787				−4.342	
Exact Monte Carlo *P*	0.15				0.008				0.007				0.0001	
99% CI	0.14 to 0.16				0.005 to 0.01				0.005 to 0.009				0 to 0.0004	

*AN* anorexia nervosa, *AN study 2* second anorexia nervosa examination, *AN study 3* third anorexia nervosa examination, *AN study 4* fourth anorexia nervosa examination, *ASD* autism spectrum disorders, *never ASD* individuals who had never been assigned an ASD diagnosis, *ASD × 1–3* those who had been assigned an ASD diagnosis in at least one study, but not at all four studies, *ASD × 4* those with an ASD diagnosis at all four examinations, *CI* confidence interval, *r × c* rows and columns.

**Table 6:** **Morgan-Russell subscale E2 (emancipation from family);** dose–response **analysis**

**Dose**	**AN study 2**				**AN study 3**				**AN study 4**				**Analysis for time trend doubly ordered r × c contingency tables Linear-by-linear association test**	**Exact Monte Carlo**
	**Response category**				**Response category**				**Response category**				**Test statistic**	***P*** **(99% CI)**
	**0**	**4**	**8**	**12**	**0**	**4**	**8**	**12**	**0**	**4**	**8**	**12**		
Never ASD	2	5	8	19	2	2	7	23	0	1	5	28	+2.562	0.01 (0.008 to 0.014)
ASD × 1-3	0	2	0	8	0	1	1	8	0	1	2	7	0	1 (0.9995 to 1)
ASD × 4	1	2	0	3	0	2	1	3	2	1	2	1	−0.743	0.54 (0.53 to 0.56)
Dose–response analysis doubly ordered r × c contingency tables	Linear-by-linear association test				Linear-by-linear association test				Linear-by-linear association test				Stratified r × c contingency tables Cochran-Mantel-Haenszel test	
Test statistic	−0.541				−0.483				−3.814				−2.606	
Exact Monte Carlo *P*	0.6				0.72				0.0005				0.01	
99% CI	0.59 to 0.62				0.71 to 0.74				0 to 0.001				0.008 to 0.013	

*AN* anorexia nervosa, *AN study 2* second anorexia nervosa examination, *AN study 3* third anorexia nervosa examination, *AN study 4* fourth anorexia nervosa examination, *ASD* autism spectrum disorders, *never ASD* individuals who had never been assigned an ASD diagnosis, *ASD × 1–3* those who had been assigned an ASD diagnosis in at least one study, but not at all four studies, *ASD × 4* those with an ASD diagnosis at all four examinations, *CI* confidence interval, *r × c* rows and columns.

**Table 7:** **Morgan-Russell subscale E5 (employment record); dose–response analysis**

**Dose**	**AN study 2**				**AN study 3**				**AN study 4**				**Analysis for time trend doubly ordered r × c contingency tables Linear-by-linear association test**	**Exact Monte Carlo**
	**Response category**				**Response category**				**Response category**				**Test statistic**	***P*** **(99% CI)**
	**0**	**4**	**8**	**12**	**0**	**4**	**8**	**12**	**0**	**4**	**8**	**12**		
Never ASD	3	3	4	24	4	1	2	27	7	2	6	19	−1.339	0.2 (0.19 to 0.21)
ASD × 1-3	3	1	0	6	0	1	4	5	5	1	0	4	−1.027	0.34 (0.33 to 0.36)
ASD × 4	3	0	0	3	4	1	0	1	4	0	1	1	−0.84	0.48 (0.46 to 0.49)
Dose–response analysis doubly ordered r × c tables	Linear-by-linear association test				Linear-by-linear association test				Linear-by-linear association test				Stratified r × c contingency tables Cochran-Mantel-Haenszel test	
Test statistic	−2				−3.22				−2.437				−4.385	
Exact Monte Carlo *P*	0.048				0.002				0.013				0	
99% CI	0.043 to 0.054				0.0008 to 0.003				0.01 to 0.016				0 to 0.0005	

*AN* anorexia nervosa, *AN study 2* second anorexia nervosa examination, *AN study 3* third anorexia nervosa examination, *AN study 4* fourth anorexia nervosa examination, *ASD* autism spectrum disorders, *never ASD* individuals who had never been assigned an ASD diagnosis, *ASD × 1–3* those who had been assigned an ASD diagnosis in at least one study, but not at all four studies, *ASD × 4* those with an ASD diagnosis at all four examinations, *CI* confidence interval, *r × c* rows and columns.

Fourth, we analysed data of the AN group in a dose–response model in relation to ASD. ‘Dose’ stands for diagnoses, ordered hierarchically into three groups: never ASD, ASD × 1–3, ASD × 4. ‘Response’ is the distribution across the existing response categories. Before any analyses were undertaken, the principal investigators hypothesised that mental, psychosexual and socioeconomic state were likely to be influenced by ASD diagnostic stability; and therefore, data was organised in ‘doubly ordered *r* × *c* contingency tables’, and consequently the relevant test was a ‘linear-by-linear association test’ [34]. The COMP group was omitted from these analyses. The results of the statistical tests are located below each study at the bottom of Tables 5, 6 and 7.

All tests were two tailed and conducted at a 5% significance level.

## Results

There was no mortality in any group at any follow-up. Any eating disorder, including AN, bulimia nervosa and eating disorder not otherwise specified, occurred in 14, 13 and 6 of the individuals in the AN group at AN study 2, 3 and 4, respectively.

### AN group versus COMP group

Generally, the findings from the stratified analysis justified the detailed statistical analyses presented here. The generalised Cochran-Mantel-Haenszel test statistic ranged from 8.7 to 48.2, which translates into exact Monte Carlo *P* values in the range 0.003 to <0.001. There were three exceptions from this general pattern: ‘attitude towards menses (if not returned)’ - almost all participants had resumed their menstruations, so there was not enough data to compute meaningful and stable figures; the scales ‘attitude to menses (if returned)’ and ‘relationship with nuclear family’ had *P* values of 0.09.

### Food intake

#### Scale A1 - dietary restriction

The distribution across categories for the variable ‘Dietary restriction’ showed inter-group difference at the first and second follow-up (AN study 2: *P* < 0.0001; AN study 3: *P* = 0.0003), and no difference at AN study 4. The change over time was due to improvement in the AN group. The dose–response analysis, that is, presence of ASD, showed that the positive development was limited to the never ASD group (significant positive trend *P* = 0.0005. Dose–response relationship was not significant at any follow-up).

#### Scale A2 - worry about body weight or appearance

The distribution across categories for the variable ‘Worry about body weight or appearance’ showed inter-group differences at all follow-ups (AN study 2: *P* < 0.0001; AN study 3: *P* < 0.0001; AN study 4: *P* = 0.018). There was no relationship between the presence of ASD and scale A2 at any follow-up.

#### Scale A3 - body weight

The difference between the AN and the COMP group was most pronounced at AN study 2 (*P* < 0.0001), still highly significant at AN study 3 (*P* = 0.0008) and non-existent at AN study 4 (*P* = 0.85). This development was the net result of a significant improvement in the AN group and an even more significant negative trend in the COMP group. The dose–response analysis showed that the positive development in body weight was limited to the never ASD group, (*P* = 0.04). There was no indication of a dose–response effect of ASD on body weight at any follow-up.

### Menstrual pattern *- scale B*

The largest between-group difference was found at AN study 2 (*P* = 0.004), a smaller difference at AN study 3 (*P* = 0.03), and no difference at AN study 4. This change over time was mainly due to a significant improvement in the AN group, and it was diminished by a similar, but not significant, tendency in the COMP group. The dose–response analysis showed that the positive trend in the AN group was limited to the never ASD group (*P* = 0.0003).

### Mental state *- scale C*

There was a highly significant between-group difference at all three follow-ups, with a dominance in the AN group giving responses in the lower categories. There was a lack of change over time (see Table 2). At AN study 3 (*P* = 0.008) and 4 (*P* = 0.007), a statistically significant, negative dose–response relationship was documented; that is, the more times ASD was present, the lower the MR scores (see Table 5).

### Psychosexual state

#### Scale D1 - attitude towards sexual matters

The responses for subscale D1 showed significant between-group differences (AN study 2: *P* = 0.005; AN study 3: *P* = 0.015; AN study 4: *P* = 0.02). A statistically significant negative dose–response relationship was evident at AN study 2 (*P* = 0.02) and 4 (*P* = 0.0004); that is, the more often ASD was assigned, the more negative attitude towards sexual matters.

#### Scale D2 - professed aims in sexual relationships

The findings for the subscale D2 showed no significant between-group differences at the three follow-ups. Dose–response analysis did not reach the level of significance at any of the three follow-ups.

#### Scale D3 - overt sexual behaviour

The distribution across the response categories for subscale D3 indicated significant between-group differences at all three follow-ups (AN study 2: *P* = 0.0007; AN study 3: *P* = 0.037; AN study 4: *P* = 0.05). The group ‘never ASD’ showed a statistically significant positive trend (*P* = 0.003). A statistically significant negative dose–response relationship was evident at AN study 2 (*P* = 0.013) and 4 (*P* = 0.0001).

#### Scale D4 - attitude to menstruation - if it has returned

The responses in subscale D4 did not indicate significant between-group differences at any point in time, and the development in the two groups over time was almost identical; no significant trend in either group. Dose–response analysis showed no significant difference at any follow-up.

#### Scale D5 - attitude to menstruation - if it has not returned

Due to few responses (AN study 2: *n* = 7; AN study 3: *n* = 7; AN study 4: *n* = 5), no differences were detected.

### Socioeconomic state

#### Scale E1 - relationship with nuclear family

The responses for subscale E1 indicated strained relationships in the AN group at the first two follow-ups. There was no difference between groups at AN study 4. There was no indication of a significant dose–response relationship at any follow-up.

#### Scale E2 - emancipation from family

The stratified analysis suggested a large difference between groups in at least one stratum. The Kruskal-Wallis test for between-group differences was significant at AN study 2 (*P* = 0.032), not significant at AN study 3 (*P* = 0.34), and highly significant at AN study 4 (*P* = 0.007) (see Table 3). The detailed analysis indicated a split in the AN-subgroups - there was a significant positive trend in the group never ASD (*P* = 0.01) but not in the two AN subgroups ASD × 1–3 and ASD × 4, respectively. A highly significant negative dose–response relationship was evident at AN study 4 (*P* = 0.0005) - the more often ASD was assigned, the less emancipation from family (see Table 3 and 6).

#### Scale E3 - personal contacts

In subscale E3, there were highly significant between-group differences at all follow-ups (AN study 2: *P* = 0.0007; AN study 3: *P* < 0.0001; AN study 4: *P* = 0.0001). The detailed dose–response analysis showed a significant negative relationship at all three follow-ups (AN study 2: *P* = 0.0004; AN study 3: *P* = 0.0045; AN study 4: *P* = 0.0003).

#### Scale E4 - social activities

This dimension of a person’s social life was severely impaired in the AN group at all follow-ups compared with the COMP group (AN study 2: *P* < 0.0001; AN study 3: *P* < 0.0001; AN study 4: *P* = 0.0015). The detailed dose–response analysis showed a significant negative dose–response relationship at all three follow-ups (AN study 2: *P* = 0.013; AN study 3: *P* = 0.005; AN study 4: *P* = 0.005).

#### Scale E5 - employment record

The employment record, as indicated in subscale E5, seemed to get progressively worse in the AN group. At AN study 2, the between-group difference was approaching the level of statistical significance; and at the next two follow-ups, the differences were clearly significant (see Table 4). The detailed dose–response analysis showed a marginally significant negative dose–response relationship at AN study 2 and a clearly significant negative dose–response relationship at AN study 3 and 4 (see Table 7).

### Self-progress rating

#### Scale G - self-progress rating

Neither time trends nor dose–response relationships were statistically significant.

## Discussion

The present study looks at the Morgan-Russell outcome data using a different approach compared to our previous studies [18-22,25]. Here, we analysed the full multinomial distribution across response categories, for all scales and subscales, at each follow-up, using exact nonparametric statistical methods [33-36]. We included analysis of a possible dose–response relationship between the presence of ASD and outcome in the AN group [34].

The major findings were 1) a clear improvement over time of the core anorectic symptoms, that is, food intake, body weight and menstrual pattern according to the MROAS in the AN group; 2) ‘Mental state’ and the psychosexual domains showed lasting problems over time in the AN group; 3) in the socioeconomic domain, the subscales ‘personal contacts’, ‘social activities’ and ‘employment record’ all showed highly significant between-group differences at all three follow-ups; 4) the presence of ASD, especially when present at all examinations, contributed to restricted outcomes in the areas mental, psychosexual and socio-economic state.

There was a distinct improvement over time of the anorectic symptoms, that is, food intake, body weight and menstrual pattern according to the MROAS in the AN group. The improvement was mainly limited to the subgroup never ASD, which constituted the majority of AN cases, corresponding to two thirds of the individuals in the AN group. Mental, psychosexual and socioeconomic state showed no improvement over time. Even though only a small minority still had an ED at follow-up, mental state showed poor results due to other psychiatric disorders in adult years, including affective disorders, ASD and anxiety disorders and obsessive-compulsive disorder in particular [22,25]. Similar psychiatric morbidity has also been reported in other long-term follow-up studies, where alcohol and substance abuse also were prevalent [6,38]. ASD, on the other hand, has only been investigated and diagnosed in follow-up studies by our group.

We have already in previous publications shown that autistic traits and ASD were overrepresented in this sample before AN onset and after recovery. Furthermore, we have shown that in the AN group, obsessive-compulsive personality traits and/or a diagnosis of ASD are predictors for a worse outcome pertaining to global assessment of functioning (GAF) [31] and Morgan-Russell average scale score [25]. This paper presents the dose–response relationship between presence of ASD and outcome according to the Morgan-Russell outcome subscales. The subscales were shown to be related to ASD; the more diagnostic stability of ASD, the worse the outcome. Earlier, ASD research has reported autistic traits dimensionally distributed in the general population [39], and that these traits have dimensional impact on overall functioning. The genetic vulnerability in AN is estimated to be at least 0.50 [40], where one subgroup consisting of 15% to 20% of AN individuals has a predisposition in terms of childhood onset autistic traits. The autistic phenotype makes these individuals more vulnerable for other psychiatric disorders, including EDs. In turn, ED or semi-starvation symptoms can also aggravate the autistic traits and lead to social isolation. The negative organic impact of the brain due to starvation may worsen the load of autism [41]. We suspect that this is the case in some of the individuals in our sample; exhibiting autistic traits in childhood and ending up with a full ASD syndrome after recovery when the core symptoms of the ED have subsided.

The degree of emancipation from family of origin was less than satisfactory in the AN group at AN study 4, 18 years after onset of AN, at age 32. The difficulties pertaining to emancipation from family has previously been elucidated by Ratnasuriya et al. [5] and Löwe et al. [6]. Our results indicate, however, that the problems with emancipation from family only occur among those who have been assigned ASD diagnoses at one or more occasions.

The findings regarding the employment record area require special attention. The largest differences between the AN and the COMP group were seen at AN study 3 and 4, when the individuals were on average 24 and 32 years old, respectively. The phenomenon probably reveals how typically developing individuals, such as in the COMP group, manage to work or study full time in this period of life, while a significant minority of the AN group is not able to adapt to adult life after quitting school. We have previously shown that at mean age 32, one in four has no paid employment due to a psychiatric disability [25]. Even though few individuals have a persisting ED 18 years after AN onset, there are still mental problems, including ASD, that result in poor employment records.

### Strengths

Our study population was community based (as opposed to a clinical population) and was matched, and data was collected in a uniform and systematic way both in the AN and the COMP group. The design was longitudinal and prospective with several follow-ups. ASD was ascertained using validated instruments, using researchers blinded to group status. Attrition was zero. The Morgan-Russell outcome assessment schedule is a well-validated and widely used instrument. Age and period effects could be studied effectively. Effects of ASD on outcome were studied in detail in a dose–response model. The statistical models and computations were compatible with the existing data structure.

### Limitations

The narrow age range - most cases were born between 1970 and 1974 - limited the possibility to study cohort effects. The study took place in one Swedish city - Gothenburg - which may limit the generalisation of findings. The relatively small cohort gave limited power to study the effect of the different predictors.

### Clinical implications

Screening for ASD symptomatology with childhood onset should be part of the initial assessment of AN patients. Treatment programmes should be modified to take account of the special needs of AN patients with ASD. This point is in agreement with conclusions by Huke et al. [17].

### Research implications

Our findings need replication, in particular by other research teams with access to population-based controlled samples would be of special interest. In this way, it might be possible to outline the ‘typical’ response of each scale or subscale at different ages in the healthy comparison cases. Possible effects of treatment may also be teased out.

## Conclusions

Outcome of teenage-onset AN is favourable with respect to mortality and persisting eating disorder, but serious problems remain in the domains ‘mental state’, ‘psychosexual function’ and ‘socioeconomic state’. Outcome is considerably worse if ASD is present. Screening for ASD should be considered in AN patients with treatment failure.

## Abbreviations

AN: anorexia nervosa
AN study 1: original study at age 16
AN study 2: 6-year follow-up at age 21
AN study 3: 10-year follow-up at age 24
AN study 4: 18-year follow-up at age 32
ASD: autism spectrum disorders
COMP: comparison group
ED: eating disorder
MROAS: Morgan-Russell outcome assessment schedule
MR scores: Morgan-Russell scores
‘never ASD’: individuals who had never been assigned an ASD diagnosis
‘ASD × 1-3’: individuals who had been assigned an ASD diagnosis in at least one study but not at all four studies
‘ASD × 4’: individuals with an ASD diagnosis at all four examinations
